# Perfil Clínico e Desfechos em 30 Dias de Pacientes Portadores de Valva Aórtica Bicúspide Submetidos à Cirurgia em Valva Aórtica e/ou Aorta

**DOI:** 10.36660/abc.20201027

**Published:** 2022-01-11

**Authors:** Marcelo Kirschbaum, Vitor Emer Egypto Rosa, Brunna Pileggi Azevedo Sampaio, Gabriela Thevenard, Nádia Romanelli Quintanilha, João Ricardo Cordeiro Fernandes, Antonio de Santis, Tarso Duenhas Accorsi, Roney Orismar Sampaio, Flavio Tarasoutchi

**Affiliations:** 1 Universidade de São Paulo Hospital das Clínicas Faculdade de Medicina Universidade de São Paulo São Paulo SP Brasil Unidade Clínica de Valvopatias - Instituto do Coração (InCor) do Hospital das Clínicas da Faculdade de Medicina da Universidade de São Paulo (HC-FMUSP), São Paulo , SP – Brasil

**Keywords:** Valva Aórtica, Cirurgia Torácica, Estenose da Valva Aórtica

## Abstract

**Fundamento:**

A válvula aórtica bicúspide (VAB) atinge de 0,5 a 2% da população e está associada a alterações valvares e de aorta. Há carência de estudos sobre o perfil desses pacientes na população brasileira.

**Objetivo:**

Descrever o perfil de pacientes com VAB submetidos à cirurgia valvar e/ou de aorta em um centro cardiológico terciário, assim como os desfechos relacionados à intervenção.

**Métodos:**

Coorte retrospectiva incluindo 195 pacientes (idade média 54±14 anos, 73,8% do sexo masculino) com diagnóstico de VAB submetidos à abordagem cirúrgica (valvar e/ou de aorta) no período de 2014 a 2019. Foram avaliados dados clínicos, ecocardiográficos e tomográficos, além das características da intervenção e eventos em 30 dias. O valor de p<0,05 foi considerado estatisticamente significante.

**Resultados:**

Encontramos alta prevalência de aneurisma de aorta (56,5%), com diâmetro médio de 46,9±10,2 mm. Insuficiência aórtica importante foi encontrada em 25,1% e estenose aórtica importante em 54,9%. Cirurgia isolada em valva aórtica foi realizada em 48,2%, cirurgia isolada de aorta em 6,7% e cirurgia combinada em 45,1%. A mortalidade em 30 dias foi de 8,2%. Na análise multivariada, os fatores preditores de desfecho combinado em 30 dias (morte, fibrilação atrial e reoperação) foram idade (OR 1,044, IC 95% 1,009-1,081, p=0,014) e o índice de massa do ventrículo esquerdo (OR 1,009, IC 95% 1,000-1,018, p=0,044).

**Conclusão:**

Pacientes com VAB abordados no nosso serviço apresentam uma maior incidência de aortopatia, com a necessidade adicional de avaliação da aorta com tomografia computadorizada ou ressonância magnética.

## Introdução

A válvula aórtica bicúspide (VAB) é a doença cardíaca congênita mais prevalente, atingindo de 0,5 a 2% da população mundial. ^1^ A expectativa de vida assemelha-se a da população geral, porém esses pacientes apresentam alterações hemodinâmicas, celulares, moleculares e genéticas que estão intrinsecamente relacionadas a repercussões na válvula aórtica e na aorta, com necessidade de intervenção cirúrgica precoce. ^4^ A prevalência e progressão destas alterações são proporcionais à idade, sendo o maior risco de eventos cardiovasculares em pacientes com idade maior que 30 anos. ^8^

Tal complexidade etiopatogênica da VAB gera uma heterogeneidade de apresentações clínicas. Além disso, há uma carência de informações sobre o perfil clínico dos pacientes portadores de VAB submetidos à cirurgia cardíaca, principalmente da população brasileira.

## Objetivo

O objetivo desse trabalho é descrever o perfil de pacientes com VAB submetidos à cirurgia valvar e/ou de aorta em um centro cardiológico terciário, além dos desfechos relacionados à intervenção.

## Métodos

População estudada: Coorte retrospectiva de pacientes acima de 18 anos com diagnóstico de VAB submetidos à abordagem cirúrgica da aorta e/ou válvula aórtica, entre os anos de 2014 a 2019. Todos os pacientes foram submetidos à ecocardiografia transtorácica e avaliação da aorta ascendente e arco aórtico por tomografia computadorizada ou ressonância magnética antes da cirurgia. A indicação cirúrgica foi baseada nos protocolos institucionais, de acordo com as diretrizes vigentes para tratamento de valvopatias e das doenças de aorta. ^9 , 10^ Foram excluídos pacientes sem documentação da avaliação de aorta ou ecocardiograma pré-procedimento. O protocolo do estudo foi revisado e aprovado pelo comitê de ética institucional local.

Protocolo do estudo: Foram avaliados dados pré-operatórios da população como idade, sexo, medicações em uso, presença de sintomas, risco cirúrgico pelo EuroSCORE II, comorbidades, características anatômicas da aorta por tomografia computadorizada ou ressonância magnética, anatomia cardíaca e valvar pelo ecocardiograma e dados laboratoriais de hemoglobina e creatinina. Nos desfechos de 30 dias, foram analisados dados de mortalidade e complicações perioperatórias, além de desfecho combinado em 30 dias de mortalidade, fibrilação atrial e reabordagem cirúrgica.

Análise estatística: Foi utilizado para análise estatística o programa SPSS versão 26 (IBM, Armonk, NY), sendo realizadas análises descritivas simples de frequência e porcentagem para variáveis categóricas, com descrição de média e desvio padrão ou mediana e intervalo interquartil para variáveis contínuas. Foi analisada a distribuição de normalidade dos dados por meio do teste de Kolmogorov-Smirnov. Para análise comparativa entre os grupos, foi utilizado teste de qui-quadrado ou teste exato de Fisher para avaliação das variáveis categóricas, conforme apropriado. Para comparação de variáveis contínuas, foi utilizado teste T de Student não pareado ou teste de Mann-Whitney, conforme apropriado. A análise univariada de preditores relacionados ao desfecho combinado em 30 dias de mortalidade, fibrilação atrial e reabordagem, foi realizada com regressão logística binária. Na análise univariada, aquelas com valor de p<0,05 foram selecionadas e inseridas no modelo multivariado de regressão logística binária. A relação da presença de estenose ou insuficiência aórtica com o índice de massa do ventrículo esquerdo foi avaliada através do método de regressão linear, sendo verificados os pressupostos necessários para o uso dessa técnica (variabilidade e distribuição dos erros). O valor de p<0,05 foi considerado estatisticamente significante.

## Resultados

**Figura 1:**
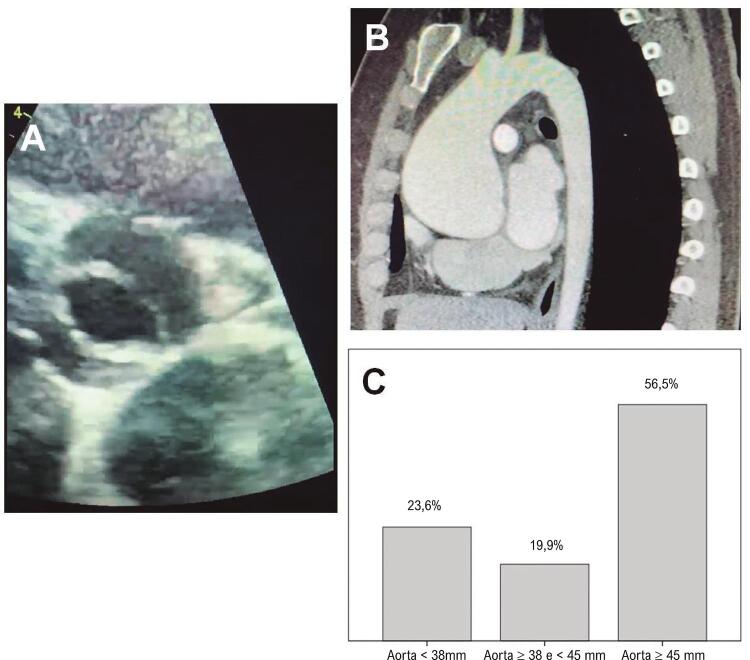
– A) Janela paraesternal transversal de ecocardiograma transtorácico evidenciando válvula aórtica bicúspide. B) Angiotomografia computadorizada de aorta evidenciando dilatação de aorta ascendente. C) Incidência de pacientes com válvula aórtica bicúspide e aorta menor que 38 mm, aorta entre 28 e 45 mm, e aorta maior ou igual a 45 mm.

Características da população: Foram incluídos 195 pacientes consecutivos portadores de VAB submetidos à cirurgia neste período. A idade média foi de 54±14 anos, maioria do sexo masculino e com alta prevalência de comorbidades como hipertensão arterial sistêmica, diabetes e doença renal crônica. As características da população estudada encontram-se na Tabela 1 . Na avaliação da aorta, 187 (95,9%) pacientes foram submetidos à realização de tomografia computadorizada e o restante (4,1%) realizaram ressonância magnética, sendo que 76,4% apresentavam ectasia aórtica (aorta > 38 mm) e 56,5% apresentavam aneurisma de aorta (aorta > 45 mm), sendo o diâmetro médio da aorta ascendente de 46,9±10,2 mm ( Figura 1 ). Pela avaliação ecocardiográfica, a média da fração de ejeção do ventrículo esquerdo pré-operatória foi de 59 ± 11%, com insuficiência aórtica importante em 25,1% e estenose aórtica importante em 54,9%. Pacientes com estenose aórtica apresentaram gradiente transaórtico médio de 49,1 ± 17,0 mmHg e área valvar aórtica de 0,79 ± 0,19 cm ^2^ . A indicação cirúrgica pela valvopatia aórtica importante ocorreu em 62,6% dos casos, 33,3% indicado por aortopatia importante e o restante por coronariopatia ou valvopatia mitral.

**Tabela 1:** – Características basais da população estudada

Variáveis	n=195
**Características clínicas**	
Idade, anos	54,7±14,1
Sexo feminino	51 (26,2)
Superfície corpórea, m ^2^	1,88±0,21
Hipertensão arterial sistêmica	111 (56,9)
Diabetes	25 (12,8)
Fibrilação atrial prévia	15 (7,7)
Doença renal crônica*	44 (22,6)
Doença arterial coronária	39 (46,7)
Endocardite prévia	9 (4,6)
Angina	46 (23,6)
Dispneia NYHA III ou IV	112 (59,1)
EuroSCORE II, %	1,61 (0,93-3,02)
Betabloqueador	90 (46,2)
Diuréticos	95 (48,7)
IECA	59 (30,3)
BRA	64 (32,8)
Estatinas	75 (38,5)
**Laboratório**	
Hemoglobina, mg/dL	13,9±1,7
Creatinina, mg/dL	1,14±0,56
**Características da aorta**	
Maior diâmetro de aorta torácica, mm	46,9±10,2
Maior diâmetro de aorta torácica indexado, mm/m ^2^	25±6
Diâmetro de aorta > 38 mm	149 (76,4)
Diâmetro de aorta > 45 mm	100 (56,5)
Dissecção aguda	11 (5,6)
Coarctação	11 (5,6)
**Ecocardiograma**	
Seio Aórtico, mm	37,4±6,8
Diâmetro do átrio esquerdo, mm	40,5±7,2
Septo, mm	12±4
Parede posterior, mm	11±1
Índice de massa de VE, g/m ^2^	142±53
Diâmetro diastólico de VE, mm	56,8±10,7
Diâmetro sistólico de VE, mm	38,3±9,4
Fração de ejeção de VE, %	59±11
Área valvar aórtica, cm ^2^ †	0,82±0,22
Gradiente transaórtico máximo, mmHg†	54±33
Gradiente transaórtico médio, mmHg†	42±19
Insuficiência aórtica importante	48 (25,1)
Estenose aórtica importante	104 (53,3)
Dupla lesão aórtica importante	16 (15,5)
**Indicação cirúrgica**	
Estenose aórtica importante	78 (40,0)
Insuficiência aórtica importante	44 (22,6)
Aorta	65 (33,3)
Coronária ou valva mitral	8 (4,1)

Dados apresentados em média ± desvio padrão, mediana (intervalo interquartil) ou n (%). *Doença renal crônica foi definida por clearance de creatinina <60ml/kg/min. †Parâmetros descritos apenas em pacientes portadores de estenose aórtica. BRA: bloqueador de receptor de angiotensina II; IECA: Inibidor da Enzima Conversora de Angiotensina; NYHA: New York Heart Association; VE: ventrículo esquerdo.

A dissecção aguda de aorta foi descrita em 5,6% dos pacientes, sendo que estes apresentavam maiores diâmetros de aorta do que os que não apresentaram dissecção aguda (54,95 ± 21,36 vs 46,81 ± 8,81 mm, p=0,010).

Características cirúrgicas e desfechos clínicos: Os dados relacionados à cirurgia e eventos clínicos estão descritos na Tabela 2 . Em 45,1% dos casos, o procedimento cirúrgico foi combinado de aorta e valva aórtica e, desses, 53,4% foi submetido à cirurgia de Bentall de Bono, 33% à cirurgia de Bentall de Bono modificada com implante de prótese biológica e os demais submetidos à cirurgia com preservação de valva aórtica. Em 94 (48,2%) pacientes foi realizada cirurgia isolada em valva aórtica e em 13 (6,7%) cirurgia isolada de aorta. Uma prótese biológica foi implantada em 60,4% dos pacientes nos quais a válvula aórtica foi abordada, prótese mecânica em 30,2%, plástica de válvula aórtica em 8,8% e 1 paciente foi submetido à abordagem transcateter (TAVI). A mortalidade em 30 dias na população foi de 8,2%, maior do que a prevista pelo EuroSCORE II (1,61 [0,93-3,02] %). No período pós-operatório, 21,5% dos pacientes apresentaram insuficiência renal aguda, 15,7% fibrilação atrial e 9,7% deles necessitaram de reoperação. Os eventos de acordo com o tipo de lesão valvar (estenose aórtica importante, insuficiência aórtica importante, dupla lesão importante e dupla lesão moderada) estão descritos na Tabela Suplementar 1.

**Tabela 2:** – Características da cirurgia e desfechos clínicos

Variável	n=195
**Procedimento**	
Cirurgia combinada (Aorta e Valva aórtica)	88 (45,1)
Bentall de Bono	47 (53,4)
Bentall de Bono modificado	29 (33,0)
Plástica valvar aórtica	12 (13,6)
Cirurgia em aorta isolada	13 (6,7)
Cirurgia em valva aórtica	94 (48,2)
Prótese biológica	80 (85,1)
Prótese mecânica	9 (9,5)
Plástica valvar	4 (4,2)
TAVI	1 (1,0)
Revascularização miocárdica associada	24 (12,3)
**Desfechos em 30 dias**	
Mortalidade	16 (8,2)
Sangramento	28 (14,4)
Hemotransfusão	41 (21)
Insuficiência renal aguda*	42 (21,5)
Reoperação	19 (9,7)
Acidente vascular cerebral	4 (2,1)
Tamponamento cardíaco	8 (4,1)
Desfecho combinado (morte + fibrilação atrial + reoperação)	55 (28,2)
Tempo de internação em UTI, dias	5,1±5,8

Dados apresentados em média ± desvio padrão ou n (%). *Insuficiência renal aguda definida como aumento de creatinina ≥ 0,3 mg/dl. TAVI: Implante de bioprótese aórtica transcateter, do inglês Transacatheter aortic valve replacement; UTI: Unidade de Terapia Intensiva.

Preditores do desfecho combinado: Os preditores do desfecho combinado em 30 dias de morte, fibrilação atrial e reoperação na análise univariada estão descritos na Tabela 3 e Tabela Suplementar 2. Na análise multivariada, idade e índice de massa do ventrículo esquerdo mantiveram-se como preditores independentes do desfecho combinado. Embora a presença de estenose ou insuficiência aórtica não seja preditora de eventos, encontramos relação dessas variáveis com o índice de massa do ventrículo esquerdo (B=18,52, IC 95%=3,96-33,09, p=0,013 e B=61,80, IC 95%=44,73-78,87, p<0,001; respectivamente). A análise multivariada excluindo o paciente submetido ao TAVI encontrou os mesmos preditores de desfecho combinado descritos anteriormente e está demonstrada na Tabela Suplementar 3.

**Tabela 3:** – Análise de preditores para o desfecho composto em 30 dias de morte, fibrilação atrial e reabordagem

Variável	Análise univariada		Análise multivariada	
	OR (IC 95%)	p	OR (IC 95%)	p
Idade, anos	1,051 (1,023-1,078)	<0,001	1,044 (1,008-1,082)	0,016
Superfície corpórea, m ^2^	0,214 (0,047-0,974)	0,046	0,178 (0,019-1,658)	0,130
Hemoglobina, mg/dL	0,812 (0,673-0,978)	0,029	0,871 (0,680-1,116)	0,276
Bloqueador de receptor de angiotensina II	1,916 (1,003-3,660)	0,049	0,680 (0,297-1,557)	0,362
Diâmetro de átrio esquerdo, mm	1,078 (1,028-1,131)	0,002	1,072 (0,995-1,155)	0,067
Índice de massa, g/m ^2^	1,007 (1,001-1,014)	0,017	1,009 (1,000-1,018)	0,044
Fração de ejeção do VE, %	0,960 (0,933-0,987)	0,004	0,981 (0,945-1,018)	0,305
Insuficiência tricúspide moderada ou importante	6,550 (1,923-22,309)	0,003	0,528 (0,095-2,950)	0,467
Insuficiência mitral moderada ou importante	2,603 (1,035-6,549)	0,042	2,646 (0,633-11,069)	0,183
Cirurgia em valva aórtica	3,257 (1,042-10,175)	0,042	2,972 (0,505-17,504)	0,229

*OR: odds ratio; VE: ventrículo esquerdo.*

Comparação de acordo com a indicação de intervenção: A comparação dos pacientes de acordo com indicação de cirurgia pelo diâmetro da aorta ou indicação pela valvopatia encontra-se na Tabela 4 . Pacientes em que a intervenção foi indicada pela doença da aorta eram menos sintomáticos e com menor remodelamento cardíaco, com índice de massa de VE menor, menor diâmetro de átrio esquerdo, septo e parede posterior menos espessos. Conforme esperado, os pacientes com indicação pela doença da aorta apresentaram maiores diâmetros de aorta e diâmetros indexados de aorta. Os pacientes indicados pela valvopatia apresentaram maior proporção de cirurgia combinada. Não encontramos diferenças entre os grupos em relação a desfechos.

**Tabela 4:** – Comparação dos pacientes de acordo com indicação de intervenção pelo diâmetro da aorta ou pela doença valvar

Variáveis	Indicação pelo diâmetro da aorta (n=65)	Indicação pela valvopatia (n=130)	p
**Características clínicas**			
Idade, anos	57,3±14,5	53,4±13,8	0,072
Superfície Corpórea, m ^2^	1,88±0,22	1,88±0,21	0,917
Sexo feminino	14 (21,5)	37 (28,5)	0,300
Hipertensão arterial sistêmica	43 (66,2)	68 (52,3)	0,066
Diabetes mellitus	8 (12,2)	17 (13,1)	0,880
Dislipidemia	21 (32,3)	37 (28,5)	0,580
Doença renal crônica*	20 (30,8)	24 (18,5)	0,053
EuroSCORE II, %	1,96 (0,97-4,43)	1,35 (0,89-2,66)	0,045
**Laboratório**			
Hemoglobina, mg/dl	14,0±1,6	13,8±1,7	0,395
Creatinina, mg/dl	1,23±0,82	1,10±0,36	0,132
**Sintomas**			
Angina	13 (20)	33 (25,4)	0,520
Dispneia NYHA III e IV	23 (35,4)	89 (68,4)	<0,001
**Medicações**			
Betabloqueador	42 (64,6)	48 (36,9)	<0,001
IECA	16 (24,6)	43 (33,1)	0,250
BRA	24 (36,9)	40 (30,8)	0,349
Espironolactona	2 (3,1)	18 (13,8)	0,020
Diurético de alça	27 (41,5)	68 (52,3)	0,185
**Característica da Aorta**			
Maior diâmetro da aorta	53,6±11,1	43,1±7,4	<0,001
Maior diâmetro da aorta indexado	28,7±6,9	23,0±4,8	<0,001
**Ecocardiograma**			
Seio Aórtico, mm	41,0±7,1	35,7±6,0	<0,001
Diâmetro do átrio esquerdo, mm	38,9±6,7	41,2±7,3	0,035
Septo, mm	11,0±1,7	12,3±4,8	0,012
Parede posterior do VE, mm	10,1±1,5	11,0±1,9	0,001
Índice de massa de VE, g/m ^2^	126,1±44,7	150,8±55,3	0,002
Diâmetro diastólico do VE, mm	54±9	57±11	0,064
Diâmetro sistólico do VE, mm	36±8	39±9	0,136
Fração de ejeção do VE, %	60±8	58±12	0,089
Gradiente transaórtico médio, mmHg	34±18	44±18	0,019
Área valvar aórtica, cm ^2^	0,91±0,27	0,80±0,20	0,137
Estenose aórtica importante	18 (27,7)	86 (66,2)	<0,001
Insuficiência Aórtica importante	10 (15,4)	38 (29,2)	0,047
Insuficiência Tricúspide moderada ou importante	3 (4,6)	10 (7,7)	0,554
Insuficiência Mitral moderada ou importante	3 (4,6)	18 (13,8)	0,059
**Cirurgia**			
Aorta isolada	13 (20)	0 (0)	<0,001
Valva aórtica isolada	0 (0)	95 (73,1)	<0,001
Cirurgia combinada	52 (80)	35 (26,9)	<0,001
**Desfecho em 30 dias**			
Morte	5 (7,7)	11 (8,5)	0,854
Fibrilação atrial pós-operatório	8 (13,6)	23 (17,6)	0,388
Reabordagem	8 (12,3)	11 (8,5)	0,403
Desfecho combinado (morte + fibrilação atrial + reoperação)	17 (26,2)	38 (29,2)	0,653

Dados apresentados em média ± desvio padrão, mediana (intervalo interquartil) ou n (%). *Doença renal crônica foi definida por clearance de creatinina <60 ml/kg/min. BRA: bloqueador de receptor de angiotensina II; IECA: Inibidor da Enzima Conversora de Angiotensina; NYHA: New York Heart Association; VE: ventrículo esquerdo.

## Discussão

Os principais achados desse estudo foram: (1) 76,4% dos pacientes com VAB apresentavam aortopatia associada, (2) por ser um centro terciário, ressalta–se uma elevada morbidade, com 56,9% de hipertensos e 46,7% de pacientes com doença arterial coronariana, deste modo encontramos uma mortalidade na intervenção maior do que a prevista pelo EuroSCORE II e (3) a idade e índice de massa foram preditores do desfecho combinado de morte, fibrilação atrial e reoperação em 30 dias.

A VAB é uma alteração na embriogênese da valva aórtica não totalmente esclarecida, porém com diversas teorias acerca de sua origem, desde alteração no fluxo transvalvar fetal levando a falha na separação das cúspides, até teorias mais atuais relacionando fatores genéticos e falha de migração celular em algumas fases da embriogênese. ^11^ A fusão das cúspides leva ao turbilhonamento do fluxo valvar, predispondo, assim, à degeneração precoce da valva aórtica. O fluxo turbilhonado também é responsável por um estresse assimétrico na parede da aorta, podendo predispor à dilatação da mesma. ^4^ Além dessa alteração hemodinâmica no fluxo da via de saída, explicando a aortopatia associada à degeneração valvar, também ocorrem alterações microscópicas como a redução da fibrilina-1, disrupção da matrix, apoptose e aumento de metaloproteinases, justificando a presença de dilatação aórtica nos pacientes com função valvar inalterada. ^6 , 7 , 14^

A indicação de abordagem na VAB pode estar relacionada à valvopatia aórtica anatomicamente importante associada a sintomas ou complicadores - indicação essa semelhante à de outras valvopatias ou aortopatia propriamente dita. A indicação de abordagem de aorta varia conforme o caso. Nos pacientes com dilatação aórtica sem valvopatia, é indicado acompanhamento frequente daqueles com diâmetro de aorta maior que 45mm ou aumento de 0,3cm/ano. As diretrizes da *European Society of Cardiology* para diagnóstico e manejo de doenças de aorta de 2014 indicam abordagem para pacientes com diâmetro de aorta >55mm isoladamente e >50mm na presença de fatores de risco. ^10^ As diretrizes da *American Heart Association* não definem valor de corte específico para abordagem isolada de aorta, orientando avaliação caso a caso de pacientes com diâmetro de aorta entre 40 e 50mm. ^15^ Ambas diretrizes orientam abordagem da aorta com diâmetro > 45mm se indicada intervenção primária valvar aórtica. ^10 , 15^

Em nosso estudo, 93% dos pacientes apresentarem valvopatia com indicação de intervenção, porcentagem semelhante ao estudo de Tzemos et al (95,7%). ^8^ Já em relação à incidência de aneurisma de aorta, existe grande variabilidade na literatura que pode ser explicada, dentre outros fatores, pela extrema heterogeneidade na definição de dilatação de aorta, variando entre 40 e 45mm. ^16^ Apesar disso, a prevalência de aneurisma de aorta definido por aorta maior que 45mm, na nossa população, superou o descrito pela literatura (56,5% vs. 20-30%, respectivamente), reforçando a necessidade de avaliação da aorta com tomografia computadorizada ou ressonância magnética em todos pacientes portadores de VAB. ^8 , 19 , 20^ Nossa população apresentou alta prevalência de hipertensão arterial sistêmica, diabetes mellitus e doença arterial coronariana se comparado a outros estudos com pacientes com VAB. ^5 , 8^ Um achado relevante foi a alta incidência de dissecção aguda (5,6%), descrita na literatura entre 0,5-1% dos pacientes com VAB em diversos estudos de desfecho cirúrgico e de seguimento a longo prazo. ^8 , 14^ Em consonância com a literatura, identificamos que tais pacientes com dissecção apresentavam maiores diâmetros de aorta do que aqueles sem tal alteração (54,9 ± 21,3 vs. 46,8 ± 8,8 mm, p=0,010). ^20^

Ressalta-se que a cirurgia combinada (aorta + válvula) também não esteve associada a pior prognóstico quando comparada à cirurgia valvar isolada. Ademais, os pacientes da nossa casuística apresentaram mortalidade em 30 dias maior que a predita pelo EuroSCORE II (8,2% vs. 2,77±4,07%, respectivamente). Além do fato do EuroSCORE II não ter validação específica para a população brasileira com VAB, a elevada mortalidade ainda pode ser justificada por um viés de seleção, dado que nosso centro é de referência nacional e há uma tendência de atendimento de pacientes mais sintomáticos (24,1% em classe funcional III/IV), com maior incidência de comorbidades (46,7% com doença arterial coronária) e com maior repercussão cardíaca (média do índice de massa de ventrículo esquerdo de 142±53 g/m ^2^ ).

O aumento do índice de massa do ventrículo esquerdo e a idade foram identificados como preditores independentes de eventos pós-operatórios, sendo esta última também descrita em outras coortes observacionais de pacientes com VAB. ^8 , 19 , 21^ Tais estudos também demonstram impacto da degeneração valvar no prognóstico, o que não foi confirmado em nosso estudo na análise multivariada. Entretanto, o aumento do índice de massa do ventrículo esquerdo teve correlação com a presença de estenose e insuficiência aórtica importantes, sendo um marcador indireto da repercussão valvar nas câmaras cardíacas esquerdas.

### Limitações

A principal limitação desse estudo é inerente ao seu desenho de caráter observacional. Dessa forma, dados que poderiam influenciar negativamente o resultado cirúrgico e impactar em eventos (como tempo de circulação extracorpórea, tempo de internação hospitalar, uso de drogas vasoativas, uso de suporte circulatório, taxa de infecção, dentre outros) não estiveram disponíveis para a análise em todos os pacientes. Além disso, o seguimento de curto prazo não nos permite extrapolar nossos achados para além do período de 30 dias. Entretanto, o número de pacientes avaliados é grande para a patologia em questão, sendo a maior casuística da literatura nacional até o momento. Outro viés decorre do fato de nossa instituição ser referência para tratamento cirúrgico de pacientes com valvopatia e aortopatia, podendo assim não representar fielmente o comportamento da doença na população geral, mas nos faz entender melhor as características da patologia em uma população altamente complexa. Além disso, o curto período de inclusão (2014 a 2019) assegurou a homogeneidade de técnicas cirúrgicas e de recomendações de intervenção. Outro ponto a ser mencionado é que não foi realizada, de maneira rotineira, a análise histopatológica da aorta nos pacientes do nosso estudo. Entretanto, devido à alta associação de aortopatia com VAB, demonstrada em estudos prévios, podemos inferir que as alterações da aorta são relacionadas à valvopatia. ^6 , 7 , 14^

## Conclusão

Em pacientes com VAB abordados no nosso serviço foi identificada uma maior incidência de aortopatia que o descrito na literatura, evidenciando a heterogeneidade sindrômica da VAB e a necessidade adicional de avaliação da aorta com tomografia computadorizada ou ressonância magnética em portadores de VAB.

## * Material Suplementar

Para informação adicional, por favor, clique aqui.


