# Supplementary material for: Perfil Clínico e Desfechos em 30 Dias de Pacientes Portadores de Valva Aórtica Bicúspide Submetidos à Cirurgia em Valva Aórtica e/ou Aorta
Source: Arq Bras Cardiol. 2022 Jan 11;118(3):588–624. [Article in Portuguese] doi: 10.36660/abc.20201027 (PMC8959030; doi:10.36660/abc.20201027)
Supplement: Supplementary file 1 [file 2020-1027-portugues-suplementar.pdf]

Tabela suplementar 1. Comparação dos pacientes de acordo com lesão valvar de base, estenose aórtica importante, insuficiência aórtica importante, dupla lesão aórtica importante ou dupla lesão aórtica moderada.

**Variável**	**Estenose aórtica importante (n=90)**	**Insuficiência aórtica importante (n=32)**	**Dupla lesão aórtica importante (n=16)**	**Dupla lesão aórtica moderada (n=57)**	**p**
Morte	12 (13,3%)	1 (3,1%)	1 (6,3%)	2 (3,5%)	0,099
Sangramento	14 (15,6%)	6 (18,8%)	3 (18,8%)	5 (8,8%)	0,485
Hemotransfusão	18 (20%)	8 (25%)	4 (25%)	11 (19,3%)	0,896
Insuficiência renal aguda	23 (25,6%)	5 (15,6%)	4 (25%)	10 (17,5%)	0,529
Acidente Vascular Cerebral	2 (2,2%)	-	-	2 (3,5%)	0,475
Reoperação	6 (6,7%)	6 (8,8%)	1 (6,3%)	6 (10,5%)	0,297
Desfecho combinado	26 (28,9%)	12 (37,5%)	4 (25%)	13 (22,8%)	0,522
Tamponamento cardíaco	-	3 (9,4%)	2 (12,5%)	3 (5,3%)	**0,011**

Tabela Suplementar 2. Analise de preditores para o desfecho composto em 30 dias de morte, fibrilação atrial e reabordagem

	**Analise univariada**		**Analise multivariada**	
	**OR (IC 95%)**	**P**	**OR**	**p**
**Características gerais**				
Idade, anos	1,051 (1,023-1,078)	<0,001	1,044 (1,008-1,082)	**0,016**
Sexo feminino	0,951 (0,466-1,940)	0,889	-	-
Superfície corpórea, m^2	0,214 (0,047-0,974)	0,046	0,178 (0,019-1,658)	0,130
Hipertensão Arterial Sistêmica	0,969 (0,513-1,818)	0,921	-	-
Diabetes Mellitus	1,852 (0,773-4,419)	0,165	-	-
Dislipidemia	0,957 (0,483-1,899)	0,901	-	-
Doença Renal Crônica	1,435 (0,693-2,950)	0,326	-	-
EuroSCORE II, %	1,033 (0,960-1,111)	0,383	-	-
**Laboratório**				
Hemoglobina, mg/dL	0,812 (0,673-0,978)	0,029	0,871 (0,680-1,116)	0,276
Creatinina, mg/dL	1,456 (0,833-2,553)	0,189	-	-
**Sintomas**				
Angina	0,985 (0,473-2,054)	0,968	-	-
Dispneia NYHA 3 e 4	0,941 (0,489-1,811)	0,855	-	-
**Medicações**				
Varfarina	1,900 (0,573-6,263)	0,292	-	-
Betabloqueador	1,206 (0,643-2,264)	0,559	-	-
BCC	1,013 (0,453-2,279)	0,974	-	-
IECA	0,626 (0,303-1,280)	0,199	-	-
BRA	1,916 (1,003-3,660)	0,049	0,680 (0,297-1,557)	0,362
Espironolactona	1,116 (0,403-3,072)	0,832	-	-
Diuréticos	1,678 (0,893-3,156)	0,108	-	-
**Aorta**				
Aneurisma	1,037 (0,533-2,016)	0,915	-	-
Dissecção aguda	1,635 (0,453-5,859)	0,451	-	-
Coarctação de aorta	2,278 (0,662-7,832)	0,191	-	-
**Ecocardiograma**				

Diâmetro de Átrio Esquerdo, mm	1,078 (1,028-1,131)	0,002	1,072 (0,995-1,155)	0,067
Septo, mm	0,973 (0,873-1,079)	0,608	-	-
Parede posterior do VE, mm	1,065 (0,894-1,268)	0,482	-	-
Índice de massa de VE, g/m^2	1,007 (1,001-1,014)	0,017	1,009 (1,000-1,018)	**0,044**
Diâmetro diastólico do VE, mm	1,022 (0,993-1,053)	0,148	-	-
Diâmetro sistólico do VE, mm	1,029 (0,993-1,065)	0,094	-	-
Fração de ejeção do VE, %	0,960 (0,933-0,987)	0,004	0,981 (0,945-1,018)	0,305
Gradiente Sistólico Aórtico médio, mmHg	0,720 (0,089-5,822)	0,758	-	-
Gradiente Sistólico Aórtico máximo, mmHg	0,995 (0,975-1,016)	0,668	-	-
Insuficiência Tricúspide moderada ou importante	6,550 (1,923-22,309)	0,003	0,528 (0,095-2,950)	0,467
Insuficiência Mitral moderada ou importante	2,603 (1,035-6,549)	0,042	2,646 (0,633-11,069)	0,183
Cirurgia em valva aórtica	3,257 (1,042-10,175)	0,042	2,972 (0,505-17,504)	0,229
Cirurgia em aorta	1,163 (0,623-2,170)	0,636	-	-
Cirurgia combinada	1,653 (0,870-3,140)	0,125	-	-

*Doença renal crônica foi definida por clearance de creatinina descrito <60ml/kg/min

BCC= Bloqueador de canais de cálcio ; BRA= Bloqueador de receptor de angiotensina II ; IECA = Inibidor da Enzima Conversora de Angiotensina; NYHA = New York Heart Association; VE = Ventrículo esquerdo

Tabela Suplementar 3. Análise multivariada de preditores para o desfecho composto em 30 dias de morte, fibrilação atrial e reabordagem excluindo paciente submetido ao TAVI

Variável	Analise multivariada	
	**OR (IC 95%)**	**p**
Idade, anos	1,044 (1,009-1,082)	**0,015**
Superfície corpórea, m^2	0,147 (0,015-1,433)	0,099
Hemoglobina, mg/dL	0,882 (0,687-1,132)	0,325
Bloqueador de receptor de angiotensina II	1,502 (0,656-3,436)	0,058
Diâmetro de átrio esquerdo, mm	1,075 (0,998-1,158)	0,058
Índice de massa, g/m^2	1,009 (1,000-1,019)	**0,042**
Fração de ejeção do VE, %	0,984 (0,947-1,021)	0,391
Insuficiência tricúspide moderada ou importante	1,454 (0,241-8,771)	0,683
Insuficiência mitral moderada ou importante	0,333 (0,076-1,453)	0,143
Cirurgia em valva aórtica	0,312 (0,054-1,818)	0,195

OR = *odds ratio*; VE = Ventrículo esquerdo
