# Supplementary material for: Perfil Clínico e Desfechos em 30 Dias de Pacientes Portadores de Valva Aórtica Bicúspide Submetidos à Cirurgia em Valva Aórtica e/ou Aorta
Source: Arq Bras Cardiol. 2022 Jan 11;118(3):588–624. [Article in Portuguese] doi: 10.36660/abc.20201027 (PMC8959030; doi:10.36660/abc.20201027)
Supplement: Supplementary file 2 [file 2020-1027-ingles-suplementar.pdf]

Supplementary Table 1. Comparison of patients according to underlying valve lesion, major aortic stenosis, major aortic regurgitation, major double aortic injury, or moderate double aortic injury.

**Variable**	**Important aortic stenosis (n=90)**	**Major aortic regurgitation (n=32)**	**Double major aortic lesion (n=16)**	**Double moderate aortic lesion (n=57)**	**for**
Death	12 (13.3%)	1 (3.1%)	1 (6.3%)	2 (3.5%)	0.099
bleeding	14 (15.6%)	6 (18.8%)	3 (18.8%)	5 (8.8%)	0.485
blood transfusion	18 (20%)	8 (25%)	4 (25%)	11 (19.3%)	0.896
Acute kidney failure	23 (25.6%)	5 (15.6%)	4 (25%)	10 (17.5%)	0.529
Stroke	2 (2.2%)	-	-	2 (3.5%)	0.475
Reoperation	6 (6.7%)	6 (8.8%)	1 (6.3%)	6 (10.5%)	0.297
Combined outcome	26 (28.9%)	12 (37.5%)	4 (25%)	13 (22.8%)	0.522
cardiac tamponade	-	3 (9.4%)	2 (12.5%)	3 (5.3%)	**0.011**

Supplementary Table 2. Analysis of predictors for the composite outcome at 30 days of death, atrial fibrillation and re-approach

	univariate analysis		Multivariate analysis	
	**OR (95% CI)**	**FOR**	**OR**	**for**
**General features**				
Age years	1.051 (1.023-1.078)	<0.001	1.044 (1.008-1.082)	**0.016**
Women	0.951 (0.466-1.940)	0.889	-	-
body surface area, m2	0.214 (0.047-0.974)	0.046	0.178 (0.019-1.658)	0.130
Systemic Arterial Hypertension	0.969 (0.513-1.818)	0.921	-	-
Diabetes Mellitus	1.852 (0.773-4.419)	0.165	-	-
Dyslipidemia	0.957 (0.483-1.899)	0.901	-	-
Chronic Kidney Disease	1.435 (0.693-2.950)	0.326	-	-
EuroSCORE II, %	1.033 (0.960-1.111)	0.383	-	-
**Laboratory**				
Hemoglobin, mg/dL	0.812 (0.673-0.978)	0.029	0.871 (0.680-1.116)	0.276
Creatinine, mg/dL	1.456 (0.833-2.553)	0.189	-	-
**Symptoms**				
Angina	0.985 (0.473-2.054)	0.968	-	-
Dyspnea NYHA 3 and 4	0.941 (0.489-1.811)	0.855	-	-
**Medications**				
Warfarin	1,900 (0.573-6.263)	0.292	-	-
beta blocker	1.206 (0.643-2.264)	0.559	-	-
BCC	1.013 (0.453-2.279)	0.974	-	-
ACEi	0.626 (0.303-1.280)	0.199	-	-
BRA	1,916 (1.003-3.660)	0.049	0.680 (0.297-1.557)	0.362
Spironolactone	1,116 (0.403-3.072)	0.832	-	-
Diuretics	1,678 (0.893-3.156)	0.108	-	-
**Aorta**				
aneurysm	1.037 (0.533-2.016)	0.915	-	-
Acute dissection	1.635 (0.453-5.859)	0.451	-	-
Aortic coarctation	2.278 (0.662-7.832)	0.191	-	-
**Echocardiogram**				

Left Atrium Diameter, mm	1,078 (1.028-1.131)	0.002	1.072 (0.995-1.155)	0.067
Septum, mm	0.973 (0.873-1.079)	0.608	-	-
LV posterior wall, mm	1.065 (0.894-1.268)	0.482	-	-
VE mass index, g/m2	1.007 (1.001-1.014)	0.017	1.009 (1,000-1.018)	**0.044**
LV diastolic diameter, mm	1.022 (0.993-1.053)	0.148	-	-
LV systolic diameter, mm	1.029 (0.993-1.065)	0.094	-	-
LV ejection fraction, %	0.960 (0.933-0.987)	0.004	0.981 (0.945-1.018)	0.305
Medium Aortic Systolic Gradient, mmHg	0.720 (0.089-5.822)	0.758	-	-
Maximum Aortic Systolic Gradient, mmHg	0.995 (0.975-1.016)	0.668	-	-
Moderate or severe tricuspid regurgitation	6,550 (1,923-22,309)	0.003	0.528 (0.095-2.950)	0.467
Moderate or severe mitral regurgitation	2.603 (1.035-6.549)	0.042	2,646 (0.633-11.069)	0.183
Aortic valve surgery	3.257 (1.042-10.175)	0.042	2.972 (0.505-17.504)	0.229
Aortic surgery	1.163 (0.623-2.170)	0.636	-	-
Combined surgery	1.653 (0.870-3.140)	0.125	-	-

*Chronic kidney disease was defined by described creatinine clearance <60ml/kg/min

BCC= Calcium channel blocker; ARB=Angiotensin II receptor blocker; ACE inhibitors = Angiotensin-Converting Enzyme Inhibitor; NYHA = New York Heart Association; LV = left ventricle

Supplementary Table 3. Multivariate analysis of predictors for the composite outcome at 30 days of death, atrial fibrillation and re-approach excluding patients undergoing TAVI

**Variable**	**Multivariate analysis**	
	**OR (95% CI)**	**for**
Age years	1,044 (1.009-1.082)	**0.015**
body surface, m2	0.147 (0.015-1.433)	0.099
Hemoglobin, mg/dL	0.882 (0.687-1.132)	0.325
Angiotensin II receptor blocker	1.502 (0.656-3.436)	0.058
Left atrium diameter, mm	1.075 (0.998-1.158)	0.058
mass index, g/m2	1.009 (1,000-1.019)	**0.042**
LV ejection fraction, %	0.984 (0.947-1.021)	0.391
Moderate or severe tricuspid regurgitation	1.454 (0.241-8.771)	0.683
Moderate or severe mitral regurgitation	0.333 (0.076-1.453)	0.143
Aortic valve surgery	0.312 (0.054-1.818)	0.195

OR = odds ratio; LV = left ventricle
