# Supplementary material for: Perfil Clínico e Desfechos em 30 Dias de Pacientes Portadores de Valva Aórtica Bicúspide Submetidos à Cirurgia em Valva Aórtica e/ou Aorta
Source: Arq Bras Cardiol. 2022 Jan 11;118(3):588–624. [Article in Portuguese] doi: 10.36660/abc.20201027 (PMC8959030; doi:10.36660/abc.20201027)
Supplement: Translation

# Translation: Clinical Profile and 30-Day Outcomes of Patients with Bicuspid Aortic Valve Undergoing Aortic Valve and/or Aorta Surgery

**Authors:** Marcelo Kirschbaum, Vitor Emer Egypto Rosa, Brunna Pileggi Azevedo Sampaio, Gabriela Thevenard, Nádia Romanelli Quintanilha, João Ricardo Cordeiro Fernandes, Antonio de Santis, Tarso Duenhas Accorsi, Roney Orismar Sampaio, Flavio Tarasoutchi

**Affiliations:** 1 Brazil Unidade Clínica de Valvopatias - Instituto do Coração (InCor) do Hospital das Clínicas da Faculdade de Medicina da Universidade de São Paulo (HC-FMUSP), São Paulo, SP – Brazil

**Keywords:** Aortic Valve, Thoracic Surgery, Aortic Valve Stenosis

## Abstract

**Background:**

The bicuspid aortic valve (BAV) affects 0.5 to 2% of the population and is associated with valve and aortic alterations. There is a lack of studies on the profile of these patients in the Brazilian population.

**Objective:**

To describe the profile of patients with BAV undergoing valve and/or aortic surgery in a tertiary cardiology center, in addition to the outcomes related to the intervention.

**Methods:**

Retrospective cohort including 195 patients (mean age 54±14 years, 73.8% male) diagnosed with BAV who underwent surgical approach (valvular and/or aorta) from 2014 to 2019. Clinical data, echocardiographic and tomographic studies were evaluated, as well as characteristics of the intervention and events in 30 days. A value of p<0.05 was considered statistically significant.

**Results:**

We found a high prevalence of aortic aneurysm (56.5%), with a mean diameter of 46.9±10.2 mm. Major aortic regurgitation was found in 25.1% and major aortic stenosis in 54.9%. Isolated aortic valve surgery was performed in 48.2%, isolated aortic surgery in 6.7% and combined surgery in 45.1%. The 30-day mortality was 8.2%. In the multivariate analysis, the predictors of the combined outcome at 30 days (death, atrial fibrillation and reoperation) were age (OR 1.044, 95% CI 1.009-1.081, p=0.014) and left ventricular mass index (OR 1.009, 95% CI 1.000-1.018, p=0.044).

**Conclusion:**

Patients with BAV approached in our service have a higher incidence of aortopathy, with the additional need to evaluate the aorta with computed tomography or magnetic resonance imaging.

## Introduction

The bicuspid aortic valve (BAV) is the most prevalent congenital heart disease, affecting 0.5 to 2% worldwide.^1 - 3^Life expectancy is similar to general population, but these patients have hemodynamic, cellular, molecular and genetic changes that are intrinsically related to repercussions on the aortic valve and aorta, requiring early surgical intervention.^4 - 7^Furthermore, the prevalence and progression of these defects are proportional to age, with the greater risk of cardiovascular outcomes in patients older than 30 years.^8^

Such complex etiopathogenicity of BAV generates a heterogeneity of clinical presentations. In addition, there is a lack of information on the clinical profile of patients with BAV undergoing cardiac surgery, especially in the Brazilian population.

## Objective

This study aims to describe the profile of patients with BAV undergoing valve and/or aortic surgery in a tertiary cardiology center, in addition to the outcomes related to the intervention.

## Methods

Study population: Retrospective cohort of patients over 18 years old with a diagnosis of BAV who underwent surgical approach to the aorta and/or aortic valve between the years 2014 to 2019. All patients underwent transthoracic echocardiography analysis and evaluation of the ascending aorta and aortic arch by computed tomography or magnetic resonance imaging before surgery. The surgical indication was based on institutional protocols, following current guidelines for the treatment of valvular heart disease and aortic diseases.^9 , 10^Patients without documentation of aortic assessment or pre-procedure echocardiogram were excluded. The study protocol was reviewed and approved by the local institutional ethics committee.

Study protocol: Preoperative data of the population such as age, sex, medications in use, presence of symptoms, surgical risk by EuroSCORE II, comorbidities, anatomical characteristics of the aorta by computed tomography or magnetic resonance imaging, cardiac and valve anatomy by echocardiogram, and laboratory data on hemoglobin and creatinine were evaluated. In the 30-day outcomes, data on perioperative mortality and complications were analyzed, in addition to the 30-day composite endpoint of mortality, atrial fibrillation and surgical reoperation.

Statistical analysis: The SPSS version 26 program (IBM, Armonk, NY) was used for statistical analysis, with simple descriptive analyzes of frequency and percentage for categorical variables, with a description of mean and standard deviation or median and interquartile range for continuous variables. Data normality distribution was analyzed using the Kolmogorov-Smirnov test. For comparative analysis between groups, the chi-square test or Fisher’s exact test was used to assess categorical variables, as appropriate. For comparison of continuous variables, unpaired Student’s t-test or Mann-Whitney test was used, as appropriate. Univariate analysis of predictors related to the 30-day composite endpoint of mortality, atrial fibrillation and reoperation was performed with binary logistic regression. In the univariate analysis, those with a p-value <0.05 were selected and included in the multivariate binary logistic regression model. The relationship of the presence of aortic stenosis or regurgitation with the left ventricular mass index was evaluated using the linear regression method, and the necessary assumptions for the use of this technique were verified (variability and distribution of errors). A value of p<0.05 was considered statistically significant.

## Results

Characteristics of the population: 195 consecutive patients with BAV who underwent surgery during this period were included. The mean age was 54±14 years, mostly male and with a high prevalence of comorbidities such as systemic arterial hypertension, diabetes, and chronic kidney disease. The characteristics of the studied population are shown in Table 1 . In the assessment of the aorta, 187 (95.9%) patients underwent computed tomography, and the remainder (4.1%) underwent magnetic resonance imaging, with 76.4 % of aortic ectasia (aorta > 38 mm), and 56.5% with aortic aneurysm (aorta > 45 mm), with a mean diameter of the ascending aorta of 46.9 ± 10.2 mm ( Figure 1 ). By echocardiographic assessment, the mean preoperative left ventricular ejection fraction was 59 ± 11%, with severe aortic regurgitation in 25.1% and severe aortic stenosis in 54.9%. Patients with aortic stenosis had a mean transaortic gradient of 49.1 ± 17.0 mmHg and aortic valve area of 0.79 ± 0.19 cm^2^. Surgical indication for aortic valve disease occurred in 62.6% of the cases, 33.3% for aortopathy and the remainder for coronary artery disease or mitral valve disease.

**Table 1:** – Baseline characteristics of the studied population

Variables	n=195
**Clinical features**	
Age, years	54.7±14.1
Female Sex	51 (26.2)
Body Surface Area, m^2^	1.88±0.21
Hypertension	111 (56.9)
Diabetes	25 (12.8)
Previous atrial fibrillation	15 (7.7)
Chronic kidney Disease*	44 (22.6)
Coronary Artery Disease	39 (46.7)
Previous Endocarditis	9 (4.6)
Angina	46 (23.6)
Dyspnea NYHA III or IV	112 (59.1)
EuroSCORE II, %	1.61 (0.93-3.02)
Beta-Blocker	90 (46.2)
Diuretics	95 (48.7)
ACEi	59 (30.3)
BRA	64 (32.8)
Statins	75 (38.5)
**Laboratory**	
Hemoglobin, mg/dL	13.9±1.7
Creatinine, mg/dL	1.14±0.56
**Characteristics of the aorta**	
Larger diameter of the thoracic aorta, mm	46.9±10.2
Larger indexed thoracic aorta diameter, mm/m^2^	25±6
Aortic diameter > 38 mm	149 (76.4)
Aorta diameter > 45 mm	100 (56.5)
Acute dissection	11 (5.6)
Coarctation	11 (5.6)
**Echocardiogram**	
Aortic sinus, mm	37.4±6.8
Left atrium diameter, mm	40.5±7.2
Septum, mm	12±4
Posterior wall, mm	11±1
LV mass index, g/m^2^	142±53
LV diastolic diameter, mm	56.8±10.7
LV systolic diameter, mm	38.3±9.4
LV ejection fraction, %	59±11
Aortic valve area, cm2†	0.82±0.22
Maximum transaortic gradient, mmHg†	54±33
Mean transaortic gradient, mmHg†	42±19
Severe aortic regurgitation	48 (25.1)
Severe aortic stenosis	104 (53.3)
Severe mixed aortic disease	16 (15.5)
**Surgical indication**	
Severe aortic stenosis	78 (40,0)
Severe aortic regurgitation	44 (22,6)
Aorta	65 (33,3)
Coronary or mitral valve	8 (4,1)

Data presented as mean ± standard deviation, median (interquartile range) or n (%). *Chronic kidney disease was defined as creatinine clearance <60ml/kg/min. †Parameters described only in patients with aortic stenosis. ARB: Angiotensin II receptor blocker; ACE inhibitors: Angiotensin-Converting Enzyme Inhibitor; NYHA: New York Heart Association; LV: left ventricle.

**Figure 1:** 

Acute aortic dissection was described in 5.6% of the patients, who had larger aortic diameters than those without acute dissection (54.95 ± 21.36 vs. 46.81 ± 8.81 mm, p=0.010).

Surgical characteristics and clinical outcomes: Data related to surgery and clinical outcomes are described in Table 2 . In 45.1%, the procedure was combined aorta and aortic valve surgery. Of these, 53.4% underwent Bentall de Bono surgery, 33% underwent modified Bentall de Bono surgery with implantation of a biological prosthesis, and the remaining underwent surgery with aortic valve preservation. In 94 (48.2%) patients, isolated aortic valve surgery was performed and 13 (6.7%) patients underwent isolated aortic surgery. In the patients undergoing aortic valve surgery, biological prosthesis was implanted in 60.4%, mechanical prosthesis in 30.2%, aortic valve repair in 8.8%, and one patient underwent transcatheter approach (TAVI). The 30-day mortality was 8.2%, higher than predicted by the EuroSCORE II (1.61 [0.93-3.02] %). In the postoperative period, 21.5% of patients had acute renal failure, 15,7% had atrial fibrillation, and 9.7% required reoperation. The outcomes according to the type of valve lesion (severe aortic stenosis, severe aortic regurgitation, severe mixed aortic disease and moderate mixed aortic disease) are described in Supplementary Table 1 .

**Table 2:** – Surgery characteristics and clinical outcomes

Variable	n=195
**Procedure**	
Combined surgery (Aorta and aortic valve)	88 (45.1)
Bentall de Bono	47 (53.4)
Modified Bentall de Bono	29 (33.0)
Aortic valve repair	12 (13.6)
Isolated aorta surgery	13 (6.7)
Aortic valve surgery	94 (48.2)
Biological prosthesis	80 (85.1)
Mechanical prosthesis	9 (9.5)
Valve repair	4 (4.2)
TAVI	1 (1.0)
Combined myocardial revascularization	24 (12.3)
**Outcomes in 30 days**	
Mortality	16 (8.2)
Bleeding	28 (14.4)
Blood transfusion	41 (21)
Acute kidney injury*	42 (21.5)
Reoperation	19 (9.7)
Stroke	4 (2.1)
Cardiac tamponade	8 (4.1)
Combined outcome (death + atrial fibrillation + reoperation)	55 (28.2)
Length of stay in the ICU, days	5.1±5.8

Data presented as mean ± standard deviation or n (%). *Acute kidney injury defined as an increase in creatinine ≥0.3 mg/dl. TAVI:Transcatheter aortic bioprosthesis implantation; ICU: Intensive Care Unit.

Predictors of the Composite Endpoint: The univariate analysis of predictors of 30-day composite endpoint of death, atrial fibrillation and reoperation are described in Table 3 and Supplementary Table 2 . In the multivariate analysis, age and left ventricular mass index remained independent predictors of the combined outcome. Although the presence of aortic stenosis or regurgitation was not predictor of endpoint, we found a relationship of these variables with the left ventricular mass index (B=18.52, 95% CI=3.96-33.09, p=0.013 and B=61.80, 95% CI=44.73-78.87, p<0.001; respectively). The multivariate analysis excluding the patient undergoing TAVI found the same composite endpoint predictors described above and is shown in Supplementary Table 3 .

**Table 3:** – Predictor analysis for the 30-day composite endpoint of death, atrial fibrillation, and re-approach

Variable	Univariate analysis		Multivariate analysis	
	OR (95% CI)	p	OR (95% CI)	p
Age, years	1.051 (1.023-1.078)	<0.001	1.044 (1.008-1.082)	0.016
Body surface area, m^2^	0.214 (0.047-0.974)	0.046	0.178 (0.019-1.658)	0.130
Hemoglobin, mg/dL	0.812 (0.673-0.978)	0.029	0.871 (0.680-1.116)	0.276
Angiotensin II receptor blocker	1,916 (1.003-3.660)	0.049	0.680 (0.297-1.557)	0.362
Left atrium diameter, mm	1,078 (1.028-1.131)	0.002	1.072 (0.995-1.155)	0.067
LV mass index, g/m^2^	1.007 (1.001-1.014)	0.017	1.009 (1,000-1.018)	0.044
LV ejection fraction, %	0.960 (0.933-0.987)	0.004	0.981 (0.945-1.018)	0.305
Moderate or severe tricuspid regurgitation	6,550 (1,923-22,309)	0.003	0.528 (0.095-2.950)	0.467
Moderate or severe mitral regurgitation	2.603 (1.035-6.549)	0.042	2,646 (0.633-11.069)	0.183
Aortic valve surgery	3.257 (1.042-10.175)	0.042	2.972 (0.505-17.504)	0.229

*OR: odds ratio; LV: left ventricle.*

Comparison according to intervention indication: The comparison of patients according to indication for surgery by aortic diameter or valve disease is shown in Table 4 . Patients in whom intervention was indicated due to disease of the aorta were less symptomatic and had less cardiac remodeling, with a smaller mass index of LV, smaller left atrium diameter, thinner septum, and posterior wall. As expected, patients with an indication for disease of the aorta had larger aortic diameters and indexed aortic diameters. Patients indicated for valvular heart disease had a higher proportion of combined surgery. We did not find differences between groups regarding outcomes.

**Table 4:** – Comparison of patients according to indication for intervention by aortic diameter or valve disease

Variables	Indication by the diameter of the aorta (n=65)	Indication for valve disease (n=130)	p
**Clinical features**			
Age, years	57.3±14.5	53.4±13.8	0.072
Body Surface Area, m^2^	1.88±0.22	1.88±0.21	0.917
Women	14 (21.5)	37 (28.5)	0.300
Hypertension	43 (66.2)	68 (52.3)	0.066
Diabetes mellitus	8 (12.2)	17 (13.1)	0.880
Dyslipidemia	21 (32.3)	37 (28.5)	0.580
Chronic kidney disease*	20 (30.8)	24 (18.5)	0.053
EuroSCORE II, %	1.96 (0.97-4.43)	1.35 (0.89-2.66)	0.045
**Laboratory**			
Hemoglobin, mg/dl	14.0±1.6	13.8±1.7	0.395
Creatinine, mg/dl	1.23±0.82	1.10±0.36	0.132
**Symptoms**			
Angina	13 (20)	33 (25.4)	0.520
Dyspnea NYHA III and IV	23 (35.4)	89 (68.4)	<0.001
**Medications**			
Beta-blocker	42 (64.6)	48 (36.9)	<0.001
ACEi	16 (24.6)	43 (33.1)	0.250
BRA	24 (36.9)	40 (30.8)	0.349
Spironolactone	2 (3.1)	18 (13.8)	0.020
Loop diuretic	27 (41.5)	68 (52.3)	0.185
**Aortic Characteristics**			
Larger diameter of the aorta	53.6±11.1	43.1±7.4	<0.001
Larger diameter of the indexed aorta	28.7±6.9	23.0±4.8	<0.001
**Echocardiogram**			
Aortic sinus, mm	41.0±7.1	35.7±6.0	<0.001
Left atrium diameter, mm	38.9±6.7	41.2±7.3	0.035
Septum, mm	11.0±1.7	12.3±4.8	0.012
LV posterior wall, mm	10.1±1.5	11.0±1.9	0.001
LV mass index, g/m^2^	126.1±44.7	150.8±55.3	0.002
LV diastolic diameter, mm	54±9	57±11	0.064
LV systolic diameter, mm	36±8	39±9	0.136
LV ejection fraction, %	60±8	58±12	0.089
Mean transaortic gradient, mmHg	34±18	44±18	0.019
Aortic valve area, cm^2^	0.91±0.27	0.80±0.20	0.137
Severe aortic stenosis	18 (27.7)	86 (66.2)	<0.001
Severe aortic regurgitation	10 (15.4)	38 (29.2)	0.047
Moderate or severe tricuspid regurgitation	3 (4.6)	10 (7.7)	0.554
Moderate or severe mitral regurgitation	3 (4.6)	18 (13.8)	0.059
**Surgery**			
Isolated aorta	13 (20)	0 (0)	<0.001
Isolated aortic valve	0 (0)	95 (73.1)	<0.001
Combined surgery	52 (80)	35 (26.9)	<0.001
**Outcome in 30 days**			
Death	5 (7.7)	11 (8.5)	0.854
Postoperative atrial fibrillation	8 (13.6)	23 (17.6)	0.388
Reoperation	8 (12.3)	11 (8.5)	0.403
Combined outcome (death + atrial fibrillation + reoperation)	17 (26.2)	38 (29.2)	0.653

Data presented as mean ± standard deviation, median (interquartile range) or n (%). *Chronic kidney disease was defined as creatinine clearance <60 ml/kg/min. ARB: Angiotensin II receptor blocker; ACE inhibitors: Angiotensin-Converting Enzyme Inhibitor; NYHA: New York Heart Association; LV: left ventricle.

## Discussion

The main findings of this study were: (1) 76.4% of patients with BAV had associated aortopathy, (2) because it is a tertiary center, high morbidity is highlighted, with 56.9% hypertensive and 46.7% of patients with coronary artery disease, therefore, we found a higher intervention mortality than predicted by the EuroSCORE II and (3) age and left ventricular mass index were predictors of the composite endpoint of death, atrial fibrillation, and reoperation within 30 days.

BAV is a defect in embryogenesis of the aortic valve not fully clarified, but with several theories about its origin, from changes in fetal transvalvular flow leading to failure in the separation of the cusps to more current theories relating genetic factors and failure of cell migration in some phases of embryogenesis.^11 - 13^The fusion of the cusps leads to the turbulence of the valve flow, thus predisposing to early aortic valve degeneration. Turbulent flow is also responsible for asymmetrical stress on the aortic wall, which may predispose to dilation of the aorta.^4^In addition to this hemodynamic change in the outflow tract that explain the aortopathy associated with valve degeneration, microscopic changes also occur such as reduced fibrillin-1, matrix disruption, apoptosis and increased metalloproteinases justifying the presence of aortic dilatation in patients with valve function unchanged.^6 , 7 , 14^

The indication for an intervention in BAV may be related to severe aortic valve disease associated with symptoms or prognostic factors *–* an indication similar to other valve diseases or aortopathy itself. The indication for intervention in the aorta varies according to the case. In patients with aortic dilatation without valve disease, frequent follow-up is indicated for those with an aortic diameter greater than 45mm or an increase of 0.3cm/year. The 2014 European Society of Cardiology Guidelines for Diagnosis and Management of Aortic Diseases indicate an intervention for patients with aortic diameter >55mm alone and >50mm in the presence of prognostic factors.^10^The American Heart Association guidelines do not define a specific cut-off value for isolated aortic intervention, guiding a case-by-case assessment of patients with aortic diameter between 40 and 50 mm.^15^Both guidelines indicate surgery in patients with aorta diameter > 45mm if primary aortic valve intervention is indicated.^10 , 15^

In our study, 93% of patients had valve disease with an indication for intervention, a percentage similar to the study by Tzemos et al (95.7%).^8^Regarding the incidence of aortic aneurysm, there is significant variability in the literature that can be explained, among other factors, by the extreme heterogeneity in the definition of aortic dilation, ranging between 40 and 45mm.^16 - 18^Despite this, the prevalence of aortic aneurysm defined by an aorta larger than 45mm in our population exceeded that described in the literature (56.5% vs. 20-30%, respectively), reinforcing the need to assess the aorta with computed tomography or magnetic resonance in all patients with BAV.^8 , 19 , 20^In addition, our population had a high prevalence of systemic arterial hypertension, diabetes mellitus and coronary artery disease compared to other studies with patients with BAV.^5 , 8^A relevant finding was the high incidence of acute dissection (5.6%), described in the literature in 0.5-1% of patients with BAV in several surgical outcomes and long-term follow-up studies.^8 , 14^In line with the literature, we identified that patients with dissection had larger aortic diameters than those without such alteration (54.9 ± 21.3 vs. 46.8 ± 8.8 mm, p=0.010).^20^

It is noteworthy that combined surgery (aorta + valve) was not associated with a worse prognosis when compared to isolated valve surgery. Furthermore, the patients in our series had 30-day mortality higher than that predicted by the EuroSCORE II (8.2% vs. 2.77±4.07%, respectively). In addition to the fact that the EuroSCORE II does not have specific validation for the Brazilian population with BAV, the high mortality can still be justified by a selection bias, given that our center is a national reference. Furthermore, there is a tendency to care for more symptomatic patients (24.1% in functional class III/IV), with a higher incidence of comorbidities (46.7% with coronary artery disease) and with greater cardiac repercussion (left ventricular mass index mean of 142±53 g/m^2^).

Increased left ventricular mass index and age were identified as independent predictors of postoperative outcomes, the latter being also described in other observational cohorts of patients with BAV.^8 , 19 , 21^Such studies also demonstrate the impact of valve degeneration on the prognosis, which was not confirmed in our study in the multivariate analysis. However, the increase in the left ventricular mass index was correlated with significant aortic stenosis and regurgitation, being an indirect marker of valve repercussion in the left cardiac chambers.

### Limitations

The main limitation of this study is inherent to its observational design. Thus, data that could negatively influence the surgical outcome and outcomes (such as cardiopulmonary bypass time, hospital stay, use of vasoactive drugs, circulatory support, infection rate, among others) were not available for analysis in all patients. Furthermore, short-term follow-up does not allow us to extrapolate our findings beyond the 30 days. However, the number of patients evaluated is large for the pathology, being the largest sample in the national literature to date. Another bias arises from the fact that our institution is a reference for surgical treatment of patients with valve disease and aortopathy and thus may not faithfully represent the behavior of the disease in the general population. However, it makes us better understand the characteristics of the pathology in a highly complex population. In addition, the short inclusion period (2014 to 2019) ensured the homogeneity of surgical techniques and intervention recommendations. Another point to be mentioned is that the histopathological analysis of the aorta was not routinely performed in the patients in our study. However, due to the high association of aortopathy with BAV, demonstrated in previous studies, we can infer that those aortic alterations are related to valve disease.^6 , 7 , 14^

## Conclusion

In patients with BAV, we found a higher incidence of aortopathy than described in the literature, showing the syndromic heterogeneity of BAV and the need for additional assessment of the aorta with computed tomography or magnetic resonance imaging.

## * Supplemental Materials

For additional information, please, click here.
